# Posterior Reversible Encephalopathy Syndrome Following Intracranial Hypotension Due to Cerebrospinal Fluid (CSF) Leakage: A Report of Two Cases

**DOI:** 10.7759/cureus.17841

**Published:** 2021-09-09

**Authors:** Abdullah Darwish, Omar Alserihy, Zaina Brinji, Elham Rawah, Ibrahim Elsodany

**Affiliations:** 1 Department of Radiology, King Abdullah Medical City, Makkah, SAU; 2 Department of Internal Medicine, King Abdullah Medical City, Makkah, SAU

**Keywords:** posterior reversible encephalopathy syndrome (pres), intracranial hypotension, neuro radiology, neuro mri, pres

## Abstract

Posterior reversible encephalopathy syndrome (PRES) in cases of intracranial hypotension is a life-threatening condition. Early suspicion, appropriate treatment, and tight control of possible contributing factors that may facilitate PRES in cerebrospinal fluid (CSF) leak patients may bring a more favorable outcome, lowering the morbidity and mortality rate.

Two cases of PRES with features of intracranial hypotension are presented.

We also discussed the possible pathogenesis of PRES in patients with intracranial hypotension. We emphasize the importance of the early diagnosis and treatment of ICH by repairing the leakage and further prompt attention to tight blood pressure control in those patients to avoid PRES development.

## Introduction

Posterior reversible encephalopathy syndrome (PRES) is a clinical neuroradiological syndrome of heterogeneous etiologies characterized by unique neurological symptoms and radiological findings. It is considered a variant of hypertensive encephalopathy and referred to as hyperperfusion encephalopathy or reversible posterior leukoencephalopathy syndrome (RPLS). [1].

It is clinically characterized by headache, visual disturbances, seizures, and altered mental function [2]. Despite that, the precise pathophysiology of the disorder is not yet fully understood and many theories were proposed mostly wandering around autoregulation failure causing blood-brain barrier disruption.

The classic radiological findings include bilateral patchy cortical and subcortical white matter signal alteration on MR and density attenuation on CT typically at the parietooccipital regions.

Intracranial hypotension, however, is not known to be an etiology for this condition. Here we report two cases of patients who presented with radiological and clinical manifestation of intracranial hypotension complicated by PRES after being subjected to spinal surgery. The syndrome may occur due to acute severe systemic hypertension, but it also has been associated with preeclampsia/eclampsia, drug toxicity, thrombotic microangiopathies, uremic encephalopathies, and sepsis.

## Case presentation

Case 1

A 44-year-old Saudi gentleman presented to our hospital complaining of neck pain associated with radiation into both upper limbs and walking difficulty. Cervical and lumbar spine MRIs were performed; the former demonstrated compressive myelopathy subsequent to posterior disc bulge at the C5-6 level. He underwent anterior cervical discectomy and fusion and developed left side weakness on the first postoperative day. Immediate head and neck CT angiography, and brain and cervical spine MRI were performed and were negative for acute abnormality. However, the cervical spine MRI demonstrated unchanged severe spinal canal stenosis at the C5-6 level. Consequently, he underwent de-compressive laminectomy of the C3-7 level. The incidental posterior dural tear was found on the second surgical attempt which was treated by dural graft.

**Figure 1:**
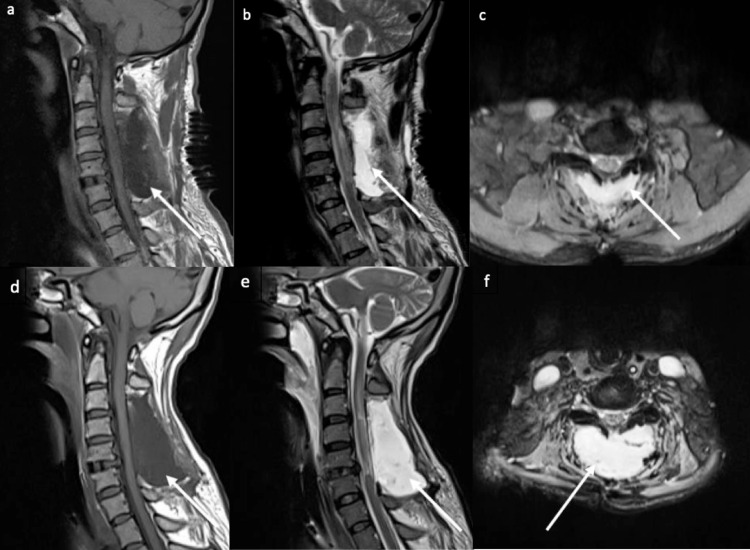
MRI of the cervical spine. Immediate post-surgical posterior decompression (a-c) demonstrates CSF-like signal collection representing a large pseudomeningocele (arrows) at the posterior para-spinal region. Follow-up cervical MRI images after two months (d-f) revealing interval increase in the size of the pseudomeningocele suggesting CSF leak. CSF - cerebrospinal fluid

About 10 days after extubation and recovery from the second surgery, the patient developed right-sided weakness and there was no improvement on the left side. So, an immediate cervical spine MRI was done and revealed sequelae of surgical intervention with C3-7 posterior decompression and anterior disk fixation at the C5-6 level. There was an associated Cerebrospinal fluid (CSF)-like signal intensity, likely pseudomeningocele in the posterior para-spinal region (Figures 1A-1C).

A few days later, he was transferred to the ICU due to hypotension and respiratory distress. He was diagnosed with H1N1 pneumonia and two days later the patient developed acute respiratory distress syndrome (ARDS). Moreover, during the management of ARDS, he had acute kidney injury and abnormal hepatic enzymes three weeks later; however, the abdominal ultrasound was normal.

**Figure 2:**
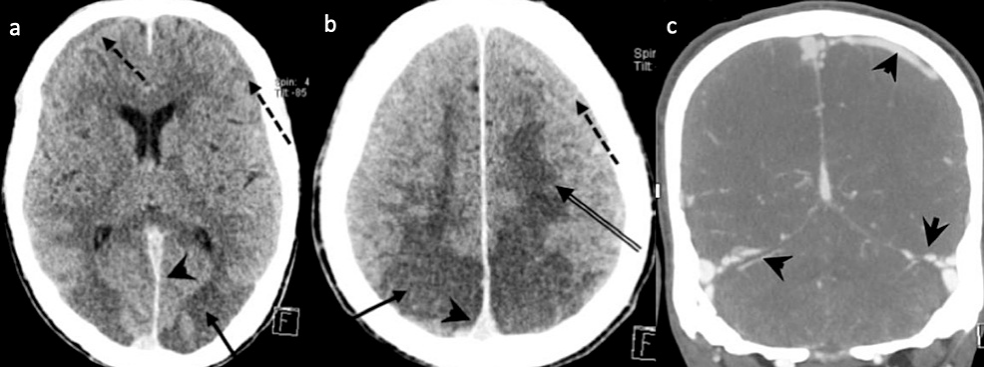
Non-enhanced CT of the brain. (a, b) Bilateral symmetrical white matter hypodense changes (vasogenic edema) involving parieto-occipital lobes (arrows) and bifrontal regions “superior frontal sulcus pattern” (double arrows), suggestive of posterior reversible encephalopathy syndrome (PRES). There were hyperdense-engorged dural venous sinuses (arrowheads) and bilateral subdural effusion (dashed arrows) as sequelae of intracranial hypotension. (c) CT venogram showing engorged dural venous sinuses and cortical veins (arrowheads).

**Figure 3:**
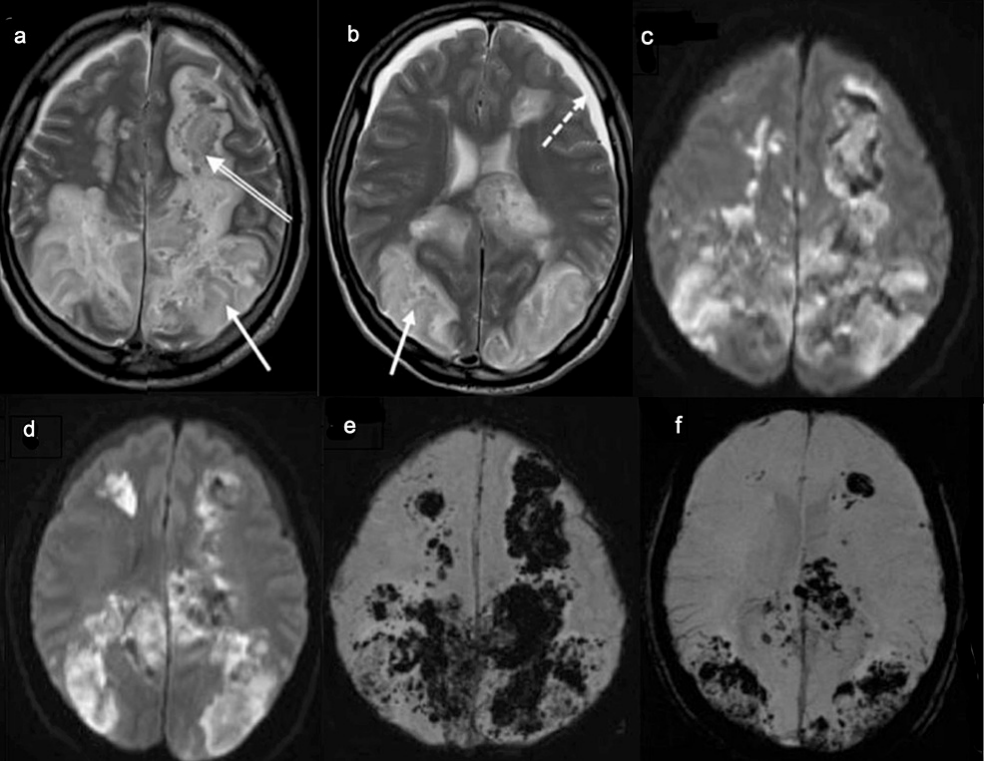
MRI of the Brain. Symmetrical bilateral white matter high T2-signal changes (a, b) with restricted diffusion on DWI (c, d) and magnetic susceptibility artifacts on SWI (e, f) in keeping with vasogenic edema with hemorrhagic changes, involving the parieto-occipital lobes (arrows), bifrontal regions “superior frontal sulcus pattern”(double arrows) and bilateral subdural effusion (dashed arrows). Features are suggestive of posterior reversible encephalopathy syndrome (PRES) complicated by lobar hemorrhage. These features were not seen in the prior exam (not shown). DWI - diffusion-weighted imaging, SWI - Susceptibility-weighted imaging

**Figure 4:**
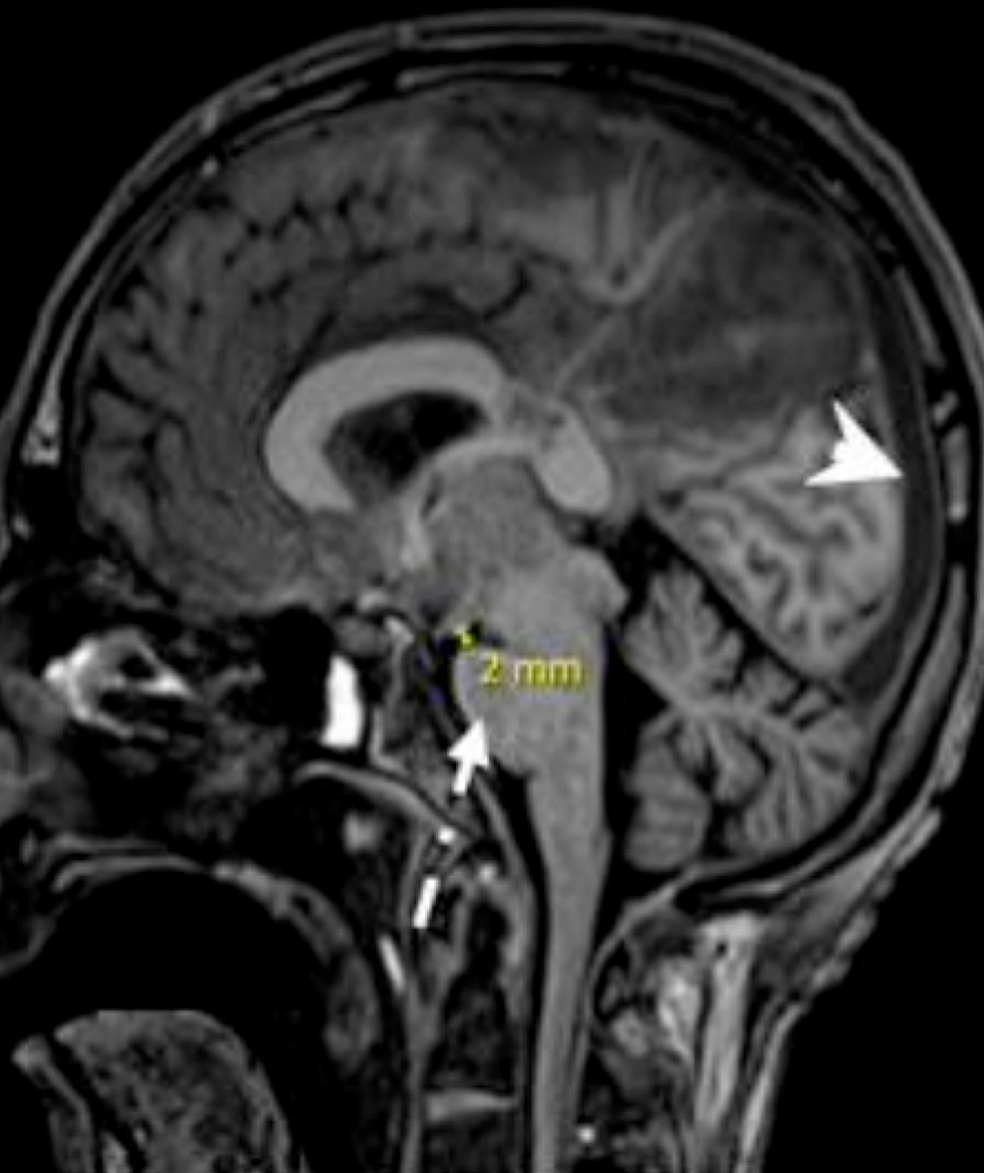
Sagittal T1 demonstrates engorged dural venous sinuses (arrowhead), flattening of the belly of the pons (dash-dot arrow), and reduced mammilopontine angle = 2 mm. These findings represent sequelae of intracranial hypotension.

In the meanwhile, the patient had attacks of seizure. CT and MRI of the brain were done (Figures 2-4) and showed extensive, symmetrical, bilateral vasogenic edema, predominately at the posterior circulation (parieto-occipital lobes), involving the bifrontal regions and associated with hemorrhagic changes (Figures 2A-2C). Features were suggestive of PRES. There was engorgement of the dural venous sinuses, bilateral subdural effusion, and sagging of the brain stem (Figures 2C, 3B, 4), suggestive of intracranial hypotension. Follow-up cervical MRI images two days later revealed interval progression and an increase in the size of the pseudomeningocele (Figures 1D-1F), suggesting CSF leak.

Unfortunately, a week later and about two months after his first surgery, the patient passed away after developing multiorgan failure, peptic ulcer with uncontrolled bleeding, septic shock due to H1N1 pneumonia, and disseminated intravascular coagulation (DIC).

Case 2

**Figure 5:**
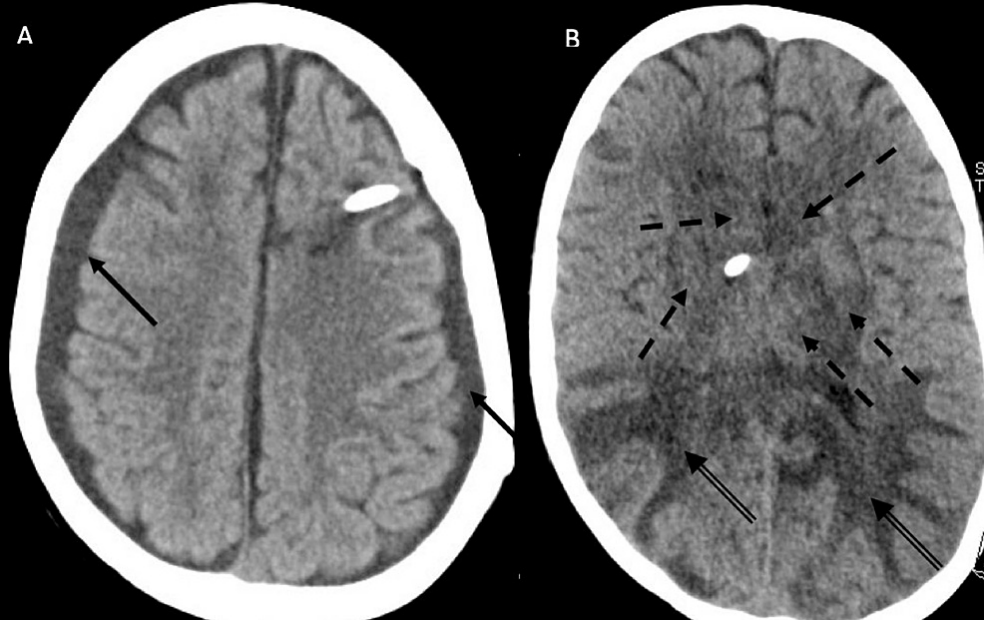
Non-enhanced CT scan of the brain. (a) Bilateral subdural effusion (arrows) secondary to over shunting. (b) Follow-up images revealed interval development of bilateral predominately parieto-occipital white matter hypodensities (double arrows) suggestive of PRES, poor differentiation of the basal ganglia and thalami with discrete hypodensities (dashed arrows), concerning hypoxic-ischemic encephalopathy, collapsed ventricular system from over shunting.

**Figure 6:**
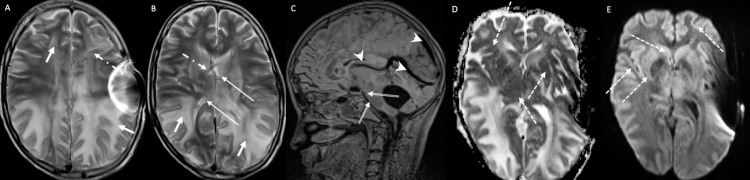
MRI of the brain. (a, b) Bilateral parieto-occipital, temporal, and bifrontal high T2 white matter signal changes (short arrows) in keeping with posterior reversible encephalopathy syndrome (PRES). The tip of the left frontal approach EVD (dash-dot arrows) is seen within the right lateral ventricle associated with the collapsed ventricular system (long-arrow) in keeping with over shunting. (c) Sagittal FLAIR demonstrates engorged dural venous sinuses and deep cerebral veins (arrowheads), flatted belly of the pons, and reduced mammilopontine angle = 1 mm (double arrows), these findings represent sequelae of intracranial hypotension secondary to over shunting. (d, e) Diffuse cerebral cortical and deep ganglionic restricted diffusion (dashed arrows) suggestive of hypoxic-ischemic encephalopathy. EVD - extra-ventricular shunt drain

A 15-year-old boy, a known case of familial multiple cavernous malformation syndrome had a history of right sub-occipital craniotomy and surgical resection of hemorrhagic pontine cavernoma, followed by insertion of extra-ventricular shunt drain (EVD). A few months later, EVD exchange was performed with insertion of ventriculoperitoneal shunt and anti-siphon device due to drain malfunction. Serial follow-up CT scans of the brain were performed and the last one revealed mildly decreased size of the ventricular system, bilateral subdural effusion, and engorged dural venous sinuses; sequelae of over-shunting were suggested. Over a week the patient’s symptoms and Glasgow Coma Scale (GCS) deteriorated. Follow-up CT brain showed interval development of diffuse bilateral symmetrical parieto-occipital and temporal white matter hypodense changes suggestive of PRES with slight further involvement of the bifrontal regions (Figure 5A). There was poor differentiation of the basal ganglia and thalami with discrete hyopodensities and persistent collapse of the third and lateral ventricles (Figure 5B). The previously seen subdural effusion was not visualized likely secondary to brain edema. Follow-up MRI of the brain showed bilateral diffuse parieto-occipital, temporal, and bifrontal high T2-signal changes in keeping with PRES (Figures 6A, 6B). Moreover, there were engorged dural venous sinuses and deep cerebral veins, flatted belly of the pons, and reduced mammilopontine angle (Figure 6C). Furthermore, there was MRI evidence of profound hypoxic-ischemic encephalopathy in the form of diffuse cerebral cortical and deep ganglionic restricted diffusion accompanied by high T2/FLAIR signal intensity (Figures 6D, 6E). The patient was managed conservatively by controlling the VP shunt through the anti-siphon device with subsequently improved ventricular over shunting and brain edema.

Unfortunately, the patient had TB meningitis three months after the last VP shunt revision surgery, then he was transferred from our tertiary center to a secondary hospital for completion of his management. Unfortunately, he passed away there four months later.

## Discussion

Pathophysiology

Although the exact pathophysiology of PRES is not clear, however, many theories have been implicated as potential mechanisms.

Extremely elevated blood pressure will subsequently lead to failure of cerebral blood vessels autoregulation, increased cerebral perfusion pressure (CPP), and endothelial dysfunction. This endothelial injury and increased capillary permeability will lead to brain vasogenic edema resulting in the classical PRES symptoms and radiological findings [3].

Alternatively, cerebrovascular hypoperfusion and venous constriction is the predominant change in some cases, causing elevation of capillary hydrostatic pressure, leading to interstitial edema or cerebral ischemia mediated by the blood-brain barrier (BBB) homeostasis alteration [4].

In imaging studies, vasogenic edema often involves the parietal and occipital lobes at the watershed distribution between the different cerebral vascular territories. However, if cerebral autoregulation is disturbed leading to vasoconstriction, the MRI will demonstrate diffusion coefficient increase [5,6].

Causes

The most common PRES etiologies are toxemia of pregnancy (eclampsia), high dose chemotherapy, organ transplantation, autoimmune disease, and infection [7,8]. Some theories do not relate hypertension to PRES exclusively since 20%-30% of patients with PRES have normal blood pressure [9].

For the two cases presented here, after excluding other more common central neurological pathology PRES was diagnosed based on typical presentation and imaging findings in the patient who had CSF leakage of a significant amount following cervical spine surgery.

The balance between cerebral arterial pulse pressure or systemic mean arterial pressure (MAP) and CSF pressure is essential to maintain the BBB integrity. This would explain why PRES happens in decrease CSF pressure. In other words, the decrease in ICP has the same effect as an increase in MAP as both will increase the CPP causing the clinico-radiological scenario of PRES [10-12].

It is not known what specific factors would accelerate the disease process or make certain patients more vulnerable to pressure differences than others. Our patients had significant CSF leakage due to the prior spinal surgery which resulted in decreased ICP and increased CPP.

Intracranial volume and pressure should be fixed at all times and its components are CSF, blood, and brain tissue. So, to ensure adequate CPP and prevent herniation, alteration in one constituent should be compensated by alteration of other constituents. So, we expect an increase of the intracranial blood volume mostly venous due to low compliance as a method of compensation to the ICP drop, which occurs in form of engorgement of meningeal vessels and venous sinuses.

This will result in venous stagnation and increased intracranial blood volume resulting in venous congestion and venous engorgement and dilatation. This is probably what happened with our two patients. Posterior predominant vasogenic edema, venous sinuses dilatation, dilated cortical veins, and bilateral subdural effusion.

There was no other convincing risk factor to be considered as the primary cause of PRES in our patient besides intracranial hypotension which is evident clinically and radiologically.

Imaging patterns

Typical imaging findings are bilateral symmetric vasogenic edema in vascular watershed zones in the posterior regions where the subcortical and deep white matter are mainly involved [13,14].

The typical type of edema is vasogenic in which there is grey-white matter differentiation preservation without diffusion restriction [15,16].

The parietal and occipital lobes are the typical distribution. The frontal, inferior temporal-occipital junction and the cerebellum follow them in involvement frequency [3,17].

The edema frequently completely reverses. The basal ganglia and brain stem also can be involved in PRESS in form of patchy areas of vasogenic edema [18,19].

Sometimes it presents by atypical features such as isolated changes of the frontal lobes, asymmetrical involvement, cortical lesions, areas of diffusion restriction, or hemorrhage [17,20].

## Conclusions

In this case report, we aim to prompt attention to the possible association of PRES, which can be considered a life-threatening condition, with intracranial hypotension. We emphasize the importance of early suspicion and appropriate treatment of PRES in cases of intracranial hypotension. Furthermore, we advise tight control of the other possible contributing factors that may facilitate PRES in CSF leak patients. To achieve that, strict blood pressure control, medication revision, and prompt infection treatment are advised hoping to prevent significant morbidity and mortality.
